# Breast/ovarian cancer genetic counseling: Do anxiety, depression, and health care‐related fears influence cancer worry and risk perception?

**DOI:** 10.1002/cam4.6518

**Published:** 2023-09-14

**Authors:** Anita Caruso, Gabriella Maggi, Cristina Vigna, Antonella Savarese, Laura Gallo, Lara Guariglia, Giulia Casu, Paola Gremigni

**Affiliations:** ^1^ Psychology Unit IRCCS “Regina Elena” National Cancer Institute Rome Italy; ^2^ Department of Oncology IRCCS “Regina Elena” National Cancer Institute Rome Italy; ^3^ Department of Psychology University of Bologna Bologna Italy

**Keywords:** anxiety, BRCA 1/2 genetic mutation, cancer, cancer worry, depression, family history, genetic counseling, health fears, psychological distress, risk perception

## Abstract

**Background:**

The impact of family and personal cancer history and emotional factors, such as depression and anxiety, on disease representation has received limited attention in studies investigating the development of cancer‐related worry and risk perception within the context of genetic counseling. The current study endeavors to fill this gap by exploring the extent to which depression and anxiety influence cancer worry and risk perception, and the role of health care‐related fear as potential mediator in this relationship.

**Methods:**

A sample of 178 women who underwent their first genetic counseling for breast/ovarian cancer, 52% of whom had previous cancer diagnoses, completed questionnaires assessing sociodemographic and clinical information, emotional distress in terms of anxiety and depression, cancer‐related worry, risk perception, and health care‐related fears.

**Results:**

Results of mediation analyses showed that cancer‐related worry and risk perception increased with rising levels of depression and anxiety, with health care‐related fears acting as a mediator in the relationship of depression and anxiety with cancer worry and risk perception. Covariate analysis revealed that previous cancer diagnosis increases cancer‐related worry but not risk perception, while the number of family members affected by cancer increases both outcomes.

**Conclusion:**

These findings emphasize the need for a holistic approach in genetic counseling and have implications for the clinical practice.

## Graphical abstract

The present study explores the extent to which depression and anxiety affect concern and risk perception, and the role of health care‐related fear as a potential mediator in this relationship. Results of mediation analyses showed that cancer‐related worry and risk perception increased with rising levels of depression and anxiety. Furthermore, health care‐related fear acted as a mediator in the relationship between depression and anxiety with cancer worry, as well as depression with risk perception. Covariate analysis revealed that the number of family members affected by cancer influenced risk perception, while previous cancer diagnosis and family cancer history affected cancer‐related worry.

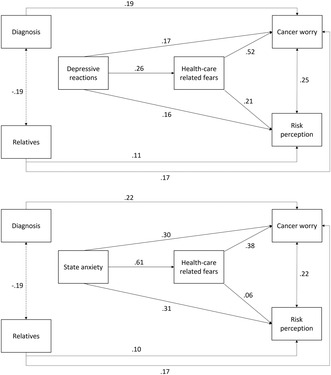

## 1 INTRODUCTION

Women diagnosed with breast and/or ovarian cancer or with a family history of these types of cancers can undergo genetic counseling to investigate whether they carry the BRCA1/2 genetic mutation. The BRCA1/2 mutation is associated with an increased risk of breast and/or ovarian cancer, with carriers of the mutation having a risk respectively of 72% and 44% for BRCA1 and 69% and 17% for BRCA2.^1^ Identification of the mutation through genetic screening and initiation of a genetic counseling process offers the opportunity to increase prophylaxis, risk reduction interventions, monitoring, and surveillance for these patients.^2^ Within genetic counseling, attention is focused not only on medical data but also on the psychosocial characteristics and issues of patients.

Women potentially carrying the BRCA1/2 genetic mutations may have a complex psychological profile, both as a result of their family members' experiences and their own illness. Scientific evidence shows that symptoms of anxiety and depression are significantly present in those undergoing genetic counseling.^3^, ^4^ Symptoms can develop in relation to being carriers of the mutation, having had direct illness experience,^5^, ^6^ having multiple family members with a history of breast/ovarian cancer,^7^ being the first tested subject in the family,^8^ and having to communicate the test outcome to their family.^9^ From the cited studies, it clearly emerges how the interaction between the individual and the family contributes to increase psychological distress. In patients carrying the genetic mutation, there are multigenerational models of the disease manifestation that can shape the development processes and have an impact in terms of psychological distress with manifestations of anxiety and depression.^10^

The family manifestation of cancer also contributes to a specific representation of the disease, such that women with family illness experiences generate representations associated with greater concern for cancer development.^11^ These data are consistent with Leventhal's self‐regulation model,^12^ which posits that the representation of illness is generated from external stimuli (witnessing a family member's illness or acquiring information from the media or doctors) and internal stimuli (direct experience of symptoms) through processes in which elements of both emotional and cognitive nature converge. The contribution of emotional factors to the genesis of the disease representation is confirmed by the works of Butler and Brand.^4^, ^13^ In their studies, the disease representation and the self‐concept, in which stigma and vulnerability dimensions converge, are associated with anxious symptomatology in these patients, showing that women with negative emotional representations present a higher level of anxiety and distress.

In the literature, it is not sufficiently clear what the relationships are between psychological distress, illness representation, risk perception, and cancer worry. The investigation of risk perception showed that these patients may have a subjective and distorted perception of risk, but it is not clear what the influence of emotional distress is in this regard. Studies that have investigated the nature of this relationship have yielded contrasting results. In Caruso et al.,^14^ there were no correlations between anxiety, depression, and risk perception. In Vos et al.'s study,^15^ physical and psychological changes, stigma, mastery, negativity, and cancer worry were correlated with risk perception not only for oneself but also for one's relatives. In Cicero et al.'s study,^16^ risk perception seems to be a moderating and/or predictive factor in the development of psychopathological symptoms, and specifically influences anxiety levels more than depression levels. In fact, in their study,^16^ it is not possible to draw definitive conclusions regarding the causal order of variables, and the directionality of significant relationships has not been determined. The study by Lerman et al.^17^ indicates the opposite, however, showing a direct influence of anxiety on risk perception. The study was conducted on patients undergoing genetic counseling and therefore adequately instructed on the actual risk probability associated with their illness condition; the results indicated that counseling on risk did not produce a better understanding in those patients who at baseline had high levels of anxious concern.^17^ Thus, anxiety seems to be a dimension that influences risk perception despite the stimuli processed at a cognitive level.

It is essential for the clinical practice to comprehend the association between family experiences, psychological distress, and risk perception. Risk perception is a relevant empirical dimension as individuals who underestimate their cancer risk are less likely to partake in health protective behaviors, while those who overestimate their risk may worry excessively and undergo unnecessary visits and checks.^18^

This study aims to investigate whether anxiety and depression affect the perception of risk and worry regarding cancer diagnosis or recurrence among those who have had cancer, and whether health care‐related fears mediate these associations. In addition, the research aims to explore how the presence/absence of the disease, considering the number of family members affected by cancer as a covariate, influences the perceived risk of carrying a genetic mutation or developing cancer and the level of cancer‐related worry.

## 2 METHODS

### 2.1 Study design and procedures

This observational, single center, cohort study focuses on women attending a specialized outpatient clinic for hereditary breast and ovarian cancer. The study considers two subgroups of the population: asymptomatic women without prior cancer diagnosis, and women already diagnosed with or having had breast or ovarian cancer, both awaiting their first genetic counseling consultation. Inclusion criteria were being 18 years or above, having at least one first‐degree relative with breast and/or ovarian cancer, having family members who have not been previously tested for the BRCA1/2 mutation, and not having undergone any genetic counseling before. Exclusion criteria consisted of women under 18 years, belonging to families already tested for BRCA1/2 diagnosis, having previously undergone genetic counseling, or waiting for a cancer screening result.

Prior to participation, potential participants were informed of the study objectives and were required to complete an informed consent form to take part in the study. Participation was completely voluntary and no compensation was offered. The study received formal ethical approval from the Ethical Committee of the “Regina Elena” National Cancer Institute (RS1721/22/2699).

### 2.2 Measures

The following data were collected: sociodemographic and clinical information, data from a self‐report questionnaire on emotional distress (anxiety, depression, and health‐related fears) and a self‐report questionnaire on cancer‐specific distress and personal and genetic risk perception.

The sociodemographic and clinical data included age, educational level, information on previous cancer diagnosis, and the number of first‐degree relatives affected by breast, ovarian, and/or other types of cancer.

### 2.3 Cognitive behavioral assessment‐hospital form

The Cognitive Behavioral Assessment‐Hospital Form (CBA‐H^19^) is a 147‐items questionnaire formed by Cards A, B, C, and D and is commonly used in the psychological assessment of patients with somatic diseases or people attending medical screening or testing. The questionnaire was validated on a sample of 4888 Italian adults formed by patients with various somatic diseases including different types of cancer (breast, ovarian, lung, and colon), individuals submitted to oncological screening, and healthy controls.^19^ Card A of the CBA‐H^19^ was used in the current study to evaluate the emotional condition of patients. It is formed by three subscales: A1–state anxiety (SA; 9 items) measures a general anxiety state; A2‐health care‐related fears (HF; 5 items) evaluates fear reactions to situations related to health management and diagnostic/curative treatments or medical procedures; and A3‐depressive reactions (DR; 5 items) investigates the presence of depressive thoughts. The items require a true/false response (coded 1‐0).

### 2.4 Cancer worry scale‐genetic counseling

The cancer worry scale was originally developed to evaluate the impact of receiving abnormal mammogram results on women's breast cancer worries, their breast self‐examination (BSE) frequency and intentions to obtain subsequent mammograms.^20^

The Italian version of the cancer worry scale–genetic counseling (CWS‐GC^21^) was modified to identify dimensions that are relevant in the genetic counseling context, such as worry about developing breast or ovarian cancer, impact of worries on daily life, and risk perception in women attending a counseling session for BRCA1/2 mutations. The CWS‐GC, used in the current study, was validated in a population of 304 Italian women, of whom 58% were diagnosed for breast or ovarian cancer and the rest were asymptomatic persons undergoing cancer genetic testing. The CWS‐GC consists of two independent indices, cancer worry (cw) and risk perception (RP). CW (five items) measures the intensity and frequency of worries about the possibility of developing cancer (or recurrence for patients who have had cancer) and the impact of worries on mood and daily functioning. Items are answered using a 5‐point scale format from 0 (not at all/never) to 4 (very much/constantly). The overall cancer‐related worries is obtained by adding and averaging the five items after transforming them into a 0–100 scale. RP (two items) measures the perceived risk of having a genetic mutation and of developing cancer (recurrently or for the first time). Both items are evaluated through a visual analogue scale ranging from “no perceived risk” (0%) to “highest perceived risk” (100%). The overall perception of risk is obtained by adding and averaging the two items.

### 2.5 Data analyses

Descriptive statistics was used to describe the sociodemographic characteristics of participants. Preliminary analyses were performed to calculate the correlations (Pearson's *r* coefficient) between the predictors, the mediator, and the dependent variables. Analysis of covariance (ANCOVA) was run to test for the effects of having had or not a previous diagnosis of cancer on the dependent variables (i.e., cancer worry and risk perception), taking into consideration the number of first‐degree relatives with cancer as a covariate.

Two mediation models were subsequently tested: Mediation model n. 1 with depressive reactions as the predictor, health care‐related fears as the mediator, and cancer worry and risk perception as the dependent variables. Mediation model n. 2 was the same as the previous one except for the predictor that was state anxiety. In the mediation models were also entered as confounder variables having had or not a previous diagnosis of cancer and the number of relatives with cancer, in the case of their significant associations with the dependent variables.

The significance level was set at *p* < 0.05. Statistical analyses were performed with the software JASP version 0.16 [2013‐2021 University of Amsterdam].

## 3 RESULTS

### 3.1 Participants

The study involved 178 women, ranging in age from 27 to 77 years old, with a mean age around 52 years. Over half of the participants had a high school qualification, one‐third held a university degree, and a minority of them had a secondary school certificate. About half of the participants had been diagnosed with cancer previously, in most cases unilateral breast cancer, while only a few of them had ovarian cancer. Finally, the number of first‐degree relatives with cancer ranged from 0 to 11, with a mean around 5. (Participants' characteristics are reported in Table 1).

**TABLE 1:** Participants' characteristics (*n* = 178).

Characteristic	Frequency (%)	Mean (standard deviation)
Age (years)	—	52.18 (10.92)
Educational level		
Secondary school	23 (12.92)	—
High school	93 (52.25)	—
University degree	62 (34.83)	—
Previous diagnosis of cancer (total)	93 (52.25)	—
Previous ovarian cancer	7 (7.53)	—
Previous breast cancer (total)	86 (92.47)	—
Unilateral	77 (89.53)	
Multiple contralateral	7 (8.14)	
Multiple ipsilateral	2 (2.33)	
Number of relatives with cancer	—	4.60 (2.49)

### 3.2 Preliminary analyses

Pearson's correlations were significant between all the psychological variables (see Table 2). In addition, the number of first‐degree relatives with cancer significantly correlated with both cancer worry (*r* = 0.21; *p* = 0.006) and risk perception (*r* = 0.28; *p* < 0.001).

**TABLE 2:** Pearson's correlations between psychological study variables.

Variable	SA	DR	HF	CW
State anxiety (SA)	—			
Depressive reactions (DR)	0.53**	—		
Health care‐related fears (HF)	0.62**	0.27**	—	
Cancer worry (CW)	0.56**	0.31**	0.57**	—
Risk perception (RP)	0.37**	0.20*	0.27**	0.49**

* *p* < 0.01

** *p* < 0.001.

ANCOVA results regarding the effects of having or not a diagnosis of cancer on cancer worry and risk perception, considering the number of relatives with cancer as a covariate, were as follows. For cancer worry, the effect of having had or not a diagnosis of cancer was significant (*F* [1,173] = 6.49, *p* = 0.01), after controlling for the effect of the number of relatives with cancer (*F* [1,173] = 10.20, *p* = 0.002) (Levene's test was nonsignificant, *p* = 0.44). Marginal means of cancer worry were 43.24 and 34.20 for having or not a cancer diagnosis, respectively, with higher worries reported by patients having had a diagnosis of cancer. For risk perception, the effects of having had or not a diagnosis was nonsignificant (*F* [1,173) = 0.40, *p* = 0.53), after controlling for the effect of the number of relatives with cancer (*F* [1,173) = 14.86, *p* < 0.001) (Levene's test was nonsignificant, *p* = 0.44). Marginal means of risk perception were 49.08 and 48.95 for having had or not a cancer diagnosis, respectively, indicating similar perception of risk between patients previously diagnosed and asymptomatic women attending the genetic counseling.

Subsequently, having had or not a cancer diagnosis and the number of relatives with cancer were both entered into the mediation model n. 1 for cancer worry, whereas only the number of relatives with cancer was entered into the mediation model n. 2 for risk perception.

### 3.3 Mediation models

**FIGURE 1:**
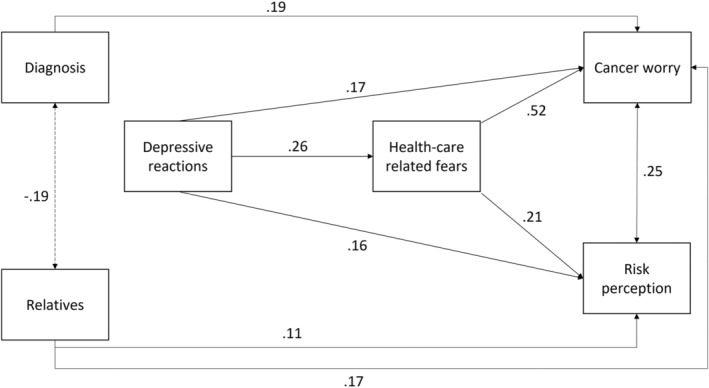
Path plot of mediation model n. 1. Diagnosis = having or not a diagnosis of cancer; Relatives = number of relatives with cancer. Standardized estimates are presented.

In the first mediation model, the predictor was depressive reactions, the dependent variables were cancer worry and risk perception, and health care‐related fears acted as a mediator. Having had or not a cancer diagnosis and the number of relatives with cancer were confounding variables for cancer worry, and only the number of relatives with cancer was a confounding variable for risk perception. The model explained 41% (*R* ^2^ = 0.41) of the variance in cancer worry and 16% (*R* ^2^ = 0.16) of the variance in risk perception. Health care‐related fears partially mediated the effect of depressive reactions on cancer worry and fully mediated the effect of depressive reactions on risk perception (see Table 3 and Figure 1). As depressive reactions increased, cancer worry and risk perception also increased, with health care‐related fears playing a partial or full mediating role, while controlling for the effects of having had or not cancer and the number of relatives with cancer.

**TABLE 3:** Parameter estimates of mediation model n. 1.

					95% Confidence interval	
Direct effects	Estimate	Std. error	z‐value	*p*	Lower	Upper
DR→CW	0.17	0.06	2.67	0.008	0.05	0.30
DR→RP	0.16	0.08	1.92	0.06	−0.004	0.33
Indirect effects						
DR→HF→CW	0.13	0.04	3.36	<0.001	0.05	0.21
DR→HF→RP	0.05	0.02	2.32	0.02	0.008	0.10
Total effects						
DR→CW	0.31	0.07	4.20	<0.001	0.16	0.45
DR→RP	0.22	0.08	2.61	0.009	0.05	0.38

*Note*: Standardized estimates, robust standard errors, robust confidence intervals, ML estimator.

Abbreviations: CW, cancer worry; DR, depressive reactions; HF, health care‐related fears; RP, risk perception.

**FIGURE 2:**
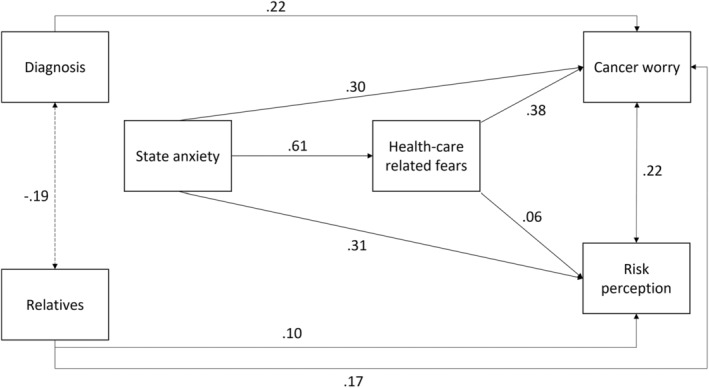
Path plot of mediation model n. 2. Diagnosis = having or not a diagnosis of cancer; Relatives = number of relatives with cancer. Standardized estimates are presented.

In the second mediation model, the predictor was state anxiety, the dependent variables were cancer worry and risk perception, and health‐care related fears acted as a mediator. Having had or not a diagnosis of cancer and the number of relatives with cancer were confounding variables for cancer worry, and only the number of relatives with cancer was a confounding variable for risk perception. The model explained 44% (*R* ^2^ = 0.44) of the variance in cancer worry and 20% (*R* ^2^ = 0.20) of the variance in risk perception. Health care‐related fears partially mediated the effect of state anxiety on cancer worry and did not mediate the effect of state anxiety on risk perception (see Table 4 and Figure 2). As state anxiety increased, cancer worry and risk Perception also increased, with health care‐related fears playing a partial mediating role for cancer worry only, while controlling for the effects of having had or not cancer and the number of relatives with cancer.

**TABLE 4:** Parameter estimates of mediation model n. 2.

					95% Confidence interval	
Direct effects	Estimate	Std. error	z‐value	*p*	Lower	Upper
SA→CW	0.30	0.09	3.41	<0.001	0.13	0.47
SA→RP	0.31	0.08	3.70	<0.001	0.15	0.48
Indirect effects						
SA→HF→CW	0.23	0.05	4.64	<0.001	0.13	0.33
SA→HF→RP	0.04	0.05	0.72	0.47	−0.06	0.14
Total effects						
SA→CW	0.53	0.07	7.78	<0.001	0.40	0.67
SA→RP	0.35	0.07	5.09	<0.001	0.21	0.48

*Note*: Standardized estimates, robust standard errors, robust confidence intervals, ML estimator.

Abbreviations: CW, cancer worry; HF, health care‐related fears; RP, risk perception; SA, state anxiety.

The comparison of the two mediation models suggests that state anxiety was a slightly superior predictor (*R* ^2^ = 0.44) of cancer worry, with the partial mediation of health care‐related fears, compared to depressive reactions (*R* ^2^ = 0.41). In terms of risk perception, state anxiety also appeared to be a better predictor (*R* ^2^ = 0.20) than depressive reactions (*R* ^2^ = 0.16). However, while the relationship between depressive reactions and risk perception was fully mediated by health care‐related fears, the relationship between state anxiety and risk perception was only direct and not mediated by health care‐related fears.

## 4 DISCUSSION

The exploration of risk perception and cancer‐related worry is a significant undertaking within the realm of psychology research. Yet, the precise mechanisms underlying these domains and the extent to which emotional and cognitive factors interplay in this respect are still shrouded in uncertainty. Current literature concerning risk perception and psychological distress is relatively scant and outdated. While contemporary studies have primarily examined how emotional and cognitive dimensions correlate, they have not delved deeply into the directionality of the associations between these variables.

The current study aimed to investigate the influence of emotional factors, such as anxiety and depression, on the perception of risk and cancer‐related worry, and to explore the mediating role of health care‐related fears in these associations. The results indicate that depression has a significant effect on both risk perception and cancer‐related worry. As the level of depression increases, so does the intensity and frequency of cancer‐related worry as well as risk perception. A previous study also found that women scoring higher in a depression scale reported higher risk estimates of developing breast cancer.^22^ Health care‐related fears play a crucial mediating role in this relationship by helping to elucidate the effect of depression on risk perception, while their role in mediating the association between depression and cancer‐related apprehension was comparatively minor.

Furthermore, the study found that state anxiety also has an influence on risk perception and cancer‐related worry. As anxiety levels increase, both cancer‐related worry and risk perception also increase. Health care‐related fears have a significant mediating role in the relationship between anxiety and cancer‐related worry, but they do not mediate the effect of anxiety on risk perception.

The findings of this study are consistent with Leventhal's self‐regulation model,^12^ which posits that individuals' perception of illness impacts their responses to it. Specifically, the perception of illness refers to how patients interpret information and personal experiences they have accumulated over time. This may elucidate the role of health care‐related fears, which mediate the relationship of depression and anxiety with risk perception and cancer‐related worry.

The present study provides evidence that anxiety is a stronger predictor of cancer‐related worry and risk perception than depression. Moreover, our findings suggest that anxiety has a direct impact on risk perception, which is not mediated by health care fears. These results are consistent with previous research conducted by Lerman,^17^ who demonstrated a correlation between anxiety and risk perception, indicating that counseling to correct risk perception is ineffective in women with frequent intrusive thoughts about the disease. Women with high levels of anxiety, according to the authors, are less likely to perceive information as reliable, as anxiety interferes with the process of attention and comprehension. Several subsequent studies have confirmed the impact of anxiety on risk perception. Cull et al.^23^ found that the best predictor of risk overestimation in a sample of women with a family history of ovarian cancer was anxiety, with their health behavior being guided by anxiety rather than objective risk. Meiser et al.^24^ found that women at increased genetic risk with higher levels of specific anxiety for cancer were more likely to overestimate the risk of ovarian cancer, especially if they had a mother diagnosed with the disease. Ultimately, the cited studies highlight the importance of considering anxiety as a key factor in cancer‐related risk perception and suggest the need for tailored interventions to reduce anxiety and prevent risk overestimation.

In order to fully examine the factors that contribute to risk perception and cancer‐related worry, we investigated the potential influence of two additional variables: the presence or absence of disease and the number of family members affected by cancer. Previous research has demonstrated that patients' family history of the disease can impact their perception of risk. Chalmers et al.^25^ found that the timing of illness and death events within a family can play a critical role in overestimating the risk. Women, in particular, may develop a sense of vulnerability through strong identification with family members affected by cancer. A similar relationship between risk perception and family history has also been identified in studies of mutation carriers aged 18–40 years.^26^, ^27^, ^28^, ^29^, ^30^

However, despite existing research, there is a lack of studies that have specifically examined how the presence or absence of cancer and the number of family members affected by cancer may impact cancer‐related worry and risk perception. Our findings suggest that the number of family members affected by cancer does have an impact on risk perception, whereas a previous cancer diagnosis does not. That is, the larger the number of family members affected by cancer the higher the perceived risk of having a genetic mutation and personally developing cancer, consistently with the literature.^25^, ^26^, ^27^, ^28^, ^29^ On the other hand, patients having had cancer and asymptomatic women attending a cancer genetic counseling manifest the same level of perceived risk of having a genetic mutation and developing cancer (recurrently or for the first time). Conversely, both variables were found to have an effect on cancer‐related worry. That is, both having had cancer and a larger number of family members affected by cancer increase the worry about developing cancer and its impact on personal daily life. It means that personal and family experiences with cancer play a critical role in increasing concern for one's personal health. These results have important implications for understanding the complex interplay of factors that contribute to perceptions of risk and worry related to cancer.

In conclusion, this study provides insights into the complex relationships between emotional factors, risk perception, and cancer‐related worry. Future research should continue to investigate these areas to better understand the underlying mechanisms and potential interventions to improve psychological well‐being in at‐risk populations.

## 5 STUDY LIMITATIONS

The present study is not without limitations, which should be acknowledged to ensure a realistic interpretation of the findings. First, the outcomes relied on self‐reported data, which are vulnerable to various biases and subjectivities. Second, the uncertainty around screening procedures may itself have induced anxiety and fear among participants, thus complicating the interpretation of results of the regression models. Additionally, patients' beliefs about the effectiveness of screening may have interacted with fear to affect their perception of risk. Third, given that the present study adopted a cross‐sectional design, it cannot infer causality, and longitudinal research in this area is warranted. Fourth, the generalizability of the findings is limited by the fact that the study's participants were highly educated women who were screened at a single institution. Therefore, caution should be exercised in generalizing the findings to other populations or screening contexts. Finally, the limited sample size of the study did not allow exploring the influence of the type of cancer previously diagnosed (breast or ovarian cancer) on the selected outcomes as well as running separate mediation models for women who had or not a diagnosis of cancer. Indeed, the number of patients who had ovarian cancer was too small (*n* = 7) to allow reliable analyses on the associations between the type of cancer and other psychological variables in the study. This study suggests that having had or not cancer influence cancer worry, so it would have been reasonable to conduct separate mediation models for the two subgroups of women (with or without a cancer diagnosis) in relation to cancer worry. However, mediation conclusions are dependent on sample size. Using a sample that is too small may pose a hindrance to adequately demonstrating the total effect. Consequently, it is advised to employ moderate sample sizes in mediation models.^31^ In our study, we deemed a sample size ranging between 100 and 200 to be moderate, while performing separate median models on the two halves of our sample, both with a sample size below 100, might not give the researchers sufficient statistical power to detect the total effect. Future research should address this issue by involving a larger number of participants, in order to reliably compare asymptomatic patients awaiting genetic screening with cancer patients, also differentiating patients based on the type of cancer using subgroups with appropriate sample sizes.

## 6 CLINICAL IMPLICATIONS

The information presented offers valuable insights for health care professionals regarding the impact that emotional well‐being may have on cognitive functioning and accurate perception of reality. In light of this, it is crucial for clinicians to adopt a holistic approach to patient care that takes into account both the physical and psychological aspects of their patients' health. To achieve this, effective communication strategies that acknowledge and respect patients' subjectivity should be prioritized while preserving their quality of life. Additionally, psychologists have a critical role to play in implementing targeted psychological interventions, including patient assessment, facilitating communication between health care teams and patients, and providing treatment when necessary.

Failure to adopt a comprehensive approach to patient care that accounts for psychological factors can lead to significant negative impacts on the decision‐making process. In particular, this may result in inappropriate allocation of medical care and diagnostic tests, as well as inadequate control over preventive therapy options. Therefore, health care providers must prioritize a patient‐centered approach that addresses broader health concerns and applies a multidisciplinary approach for optimal patient management.

## 7 CONCLUSIONS

The scenario under consideration presents a clear complexity, that can be discerned through a careful analysis of the available data. Specifically, the patients' life experiences, shaped by their own encounters with cancer or those of their family members, engender a multifaceted representation of the disease. This representation is characterized by emotional factors such as anxiety and depression, as well as health care‐related elements like fears related to surgical intervention, medical testing, and interactions with physicians. Such an interpretation of the patient's condition is further augmented by their anxiety and depression, which can exacerbate their perception of personal vulnerability in terms of risk and concerns related to cancer.

## AUTHOR CONTRIBUTIONS

**Anita Caruso:** Conceptualization (equal); data curation (equal); investigation (equal); methodology (equal); project administration (equal); resources (equal); supervision (equal); validation (equal); writing – original draft (equal); writing – review and editing (equal). **Gabriella Maggi:** Conceptualization (equal); investigation (equal); writing – original draft (equal); writing – review and editing (equal). **Cristina Vigna:** Data curation (equal); writing – review and editing (equal). **Antonella Savarese:** Writing – original draft (equal); writing – review and editing (equal). **Laura Gallo‐:** Writing – original draft (equal); writing – review and editing (equal). **Lara Guariglia:** Conceptualization (equal); investigation (equal); methodology (equal); validation (equal); writing – original draft (equal); writing – review and editing (equal). **Giulia Casu:** Data curation (equal); formal analysis (equal); investigation (equal); methodology (equal); supervision (equal); writing – original draft (equal); writing – review and editing (equal). **Paola Gremigni:** Conceptualization (equal); data curation (equal); formal analysis (lead); investigation (equal); methodology (equal); software (equal); supervision (equal); writing – original draft (equal); writing – review and editing (equal).

## FUNDING INFORMATION

This work was financially supported through funding from the institutional “Ricerca Corrente 2023.”

## CONFLICT OF INTEREST STATEMENT

The authors declare that they have no competing interests.

## Data Availability

The data that support the findings of this study are available from the corresponding author upon reasonable request.
